# Two cases of diquat poisoning in adolescent children

**DOI:** 10.1186/s13052-024-01640-x

**Published:** 2024-04-22

**Authors:** Mengtao Duan, Baowang Yang, Xiaohang Cheng, Fuhui Shen, Xia Lu, Fan Wang

**Affiliations:** 1 The Second Clinical Medical College of Lanzhou University, 199 Donggang West Road, Chengguan District, 730030 Lanzhou City, Gansu Province China; 2 Lanzhou University Second Hospital , No.82 Cuiyingmen, Linxia Road, Chengguan District, 730030 Lanzhou City, Gansu Province China

**Keywords:** Toxicology, Diquat, Pediatrics, Investigation, Blood perfusion

## Abstract

Diquat (DQ) is among the most widely used herbicides, and its intake can cause severe systemic toxicity that manifests rapidly. The resultant symptoms can cause the dysfunction of a range of tissues and organs,. As there is no specific antidote for diquat poisoning and the efficacy of extant treatments is suboptimal, physicians must acquire a more comprehensive understanding of the most effective approaches to managing affected patients. Relative few studies have been published to date focused on diquat poisoning in pediatric patients. In this report, we compare two similar cases of juvenile diquat poisoning with dynamic changes in clinical manifestations, laboratory values, and imaging results. For the first time, the difference in whether to perform blood flow perfusion and the time difference of initiation of hemoperfusion had a clear clinical difference in the subsequent effects of diquat poisoning in children with diquat poisoning. Limited evidence is available regarding the efficacy of early hemoperfusion for diquat poisoning; however, the differences in clinical outcomes articulated here highlight the benefits of early and timely hemoperfusion therapy in the treatment of DQ toxicity in children, in conjunction with primary supportive care in the management of DQ poisoning in children.

## Background

Diquat (DQ) is a non-selective, fast-acting biocidal herbicide and a paraquat co-pyridine. No antidotes are currently available to treat patients suffering from DQ poisoning, and available treatments remain limited in efficacy such that the case fatality rate remains relatively high.After 2 h of ingestion, diquat concentrations can reach peak blood levels [1]. In this report, we discuss two cases of DQ poisoning in children admitted to the Second Hospital of Lanzhou University. These children were provided with rapid treatment, but in Case B the child was not provided with hemoperfusion in a timely manner and presented with obvious abnormalities. Otherwise, the treatment path for both children was quite comparable, but the follow-up imaging and laboratory testing conducted for Case B revealed pronounced abnormalities. Studies have demonstrated that hemoperfusion effectively removes diquat from the bloodstream [2]. However, there are few reports on whether prompt and early renal support therapy (e.g., hemoperfusion) in the setting of diquat poisoning has a protective effect on organ function damage. This report provides a summary of our experiences diagnosing and treating these children, together with recommendations for the diagnosis and treatment of DQ poisoning in children in an effort to improve their prognostic outcomes.

Diquat (DQ;1,1’-ethylene-2,2’-bipyridinium ion) is a bipyridinium herbicide with the molecular formula C_12_H_12_Br_2_N_2_-H_2_O. This broad-spectrum fast-acting herbicide can non-selectively kill stems and leaves on contact. It is hydrophilic and a pesticide with moderate toxicity. When ingested, DQ can rapidly disseminate systemically through the bloodstream, accessing tissues other than the brain and spinal cord [3]. The half-life of DQ ranges from 2 days in the air to 2–10 days in the water and 3450 days in the soil [4], and exposure can cause toxicity, fetal malformation, and other forms of reproductive toxicity in mammals [5–6].

Wilks et al. classified the severity of DQ poisoning based on the ingested dose as follows: (1) Mild poisoning [ingestion of < 1 g of diquat cation, such as < 0.35mL of a 20% solution (100 g dibromide salt / 500 ml)], resulting in gastrointestinal symptoms and potential renal insufficiency that are reversible; (2) Moderate to severe poisoning (ingestion of 1–12 g of diquat cation [9.36-112.2mL]), resulting in multi-organ dysfunction characterized by prominent renal failure from which approximately two-thirds of children can recover; (3) Fulminant poisoning (ingestion of > 12 g of diquat cation, tor > 112.2mL of a commercial preparation), resulting in rapidly progressive multiple organ failure and death within 24–48 h. The World Health Organization’s International Programme on Chemical Safety defines the lethal dose of DQ as 6–12 g, and DQ poisoning-associated mortality rates are significantly positively correlated with the ingested dose.

In 2018, the European Union decided not to approve DQ and most developed nations curtailed or eliminated the use of this herbicide. However, in developing nations including China, it remains in widespread use, and cases of DQ poisoning in children are rarely reported. After the banning of paraquat in China in 2016, DQ emerged as the most prominent herbicide used in agricultural settings and the number of DQ poisoning cases rose annually. However, the Codex Committee on Pesticide Residues (CCPR) exhibited a lack of toxicological data pertaining to diquat dipyridone [7]. In patients admitted to the hospital, it is vital to assess the type and concentration of DQ in the urine or plasma. DQ poisoning cases are also typically concentrated in rural areas, however most developing countries’ county hospitals and community hospitals generally lack sufficient testing for poisons and nowadays guidelines regarding the treatment of these poisoning cases are lacking. Clear laboratory results cannot be given, and clear indicators for performing hemoperfusion are lacking.

DQ absorption can occur through the digestive or respiratory tract, or via the ocular or mucocutaneous routes, and there have also been reports of poisoning via intramuscular, subcutaneous, and vaginal contact [8–9]. While the rate of DQ absorption via the digestive tract is low, it can be rapidly distributed throughout the body and accumulate in the liver, kidney, gastrointestinal tract, and lungs, reaching peak levels within 2 h and then rapidly decreasing in concentration [4]. Approximately 90–95% of DQ is excreted in the feces in an unmodified form within 24 h, while DQ and its metabolites that are absorbed into the blood are primarily excreted in the urine within 48 h in a manner unrelated to the route of exposure [9–10]. Following poisoning, DQ can cause damage to the liver, kidneys, and lungs, in addition to causing reproductive toxicity [5]. It can even cause the onset of toxic encephalopathy [11]. However, DQ enrichment in the lungs does not occur rapidly such that there is a lag period before DQ-induced pulmonary injury caused by redox reactions [12]. DQ poisoning generates oxygen radicals that contribute to membrane lipid peroxidation and cell death. There have been reports suggesting that dynamic changes in neutrophil and white blood cell counts can help predict survival outcomes in affected patients [13], as in the hemodynamic monitoring performed for Case B in the present report.

Several different mechanisms of DQ poisoning have been proposed. For one, DQ can induce oxidative stress via the dysregulation of normal redox processes, contributing to cellular and mitochondrial dysfunction [14]. In addition, DQ can cause neurodegeneration as evidenced by the axonal degeneration and pontine demyelination evident in some patients. Although the specific mechanism is unknown [15–16], neurological damage has repeatedly been reported [17], with some studies suggesting it is associated with neurodegenerative Parkinson’s disease owing to > 80% reductions in dopamine uptake [4], as these reductions are DQ dose-dependent [14]. DQ can also induce the apoptotic death of exposed cells [14]. There is also strong evidence for the ability of DQ to cause gastrointestinal damage, with one report having demonstrated ht that the chronic intake of low DQ doses resulted in low levels of intestinal inflammation and a corresponding increase in small intestinal activity [18]. Different treatments can be administered based on the mechanistic basis for DQ poisoning. For example, N-acetylcysteine, reduced glutathione, vitamin C, melatonin, and/or melatonin can be administered to mitigate oxidative stress, while symptomatic supportive care is provided in other cases, with a clear time for the application of renal support therapy not yet having been defined.

## Case presentation

**Fig. 1:**
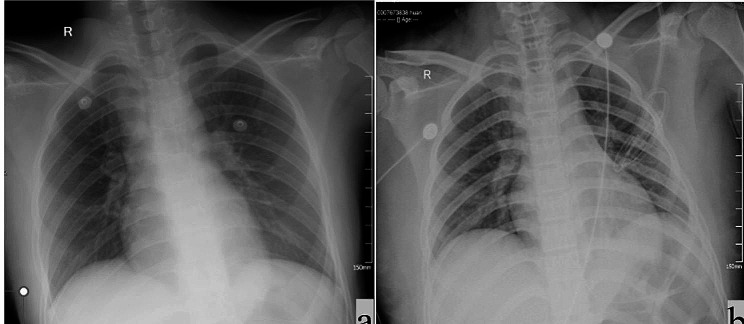
Chest orthographic radiography results: **a** Case A on the first day of admission and **b** Case B on day 5 of admission, both revealing no obvious abnormalities

Case A: A 13-year-old, previously healthy Chinese girl was sent to a local hospital 1 h after ingesting DQ. Two hours later, the patient had experienced > 10 episodes of emesis, producing pale yellow vomitus with a pungent taste. She was taken to the local Emergency Department immediately after being found to have consumed the pesticide, and she exhibited normal vital signs when first evaluated. Two hours later, she exhibited persistent burning upper abdominal pain and palpitations. She did not exhibit any drooling or respiratory symptoms, but the posterior pharynx was erythematous. After receiving a gastric lavage, she was transferred to Lanzhou University Second Hospital pediatric intensive care unit (PICU) 8 h after ingesting DQ. Her vital signs on admission to our hospital were stable. Her initial laboratory findings, including complete blood count and biochemistry results, general status, clinical symptoms, DQ ingestion, and treatment are summarized in Table 1. The results of her laboratory studies on the first day of hospitalization are summarized in Table 2. Gastroscopy results on the fourth day of admission revealed erosive esophagitis and superficial gastritis with bile reflux. Her imaging results can be seen in Fig. 1a.

**Fig. 2:**
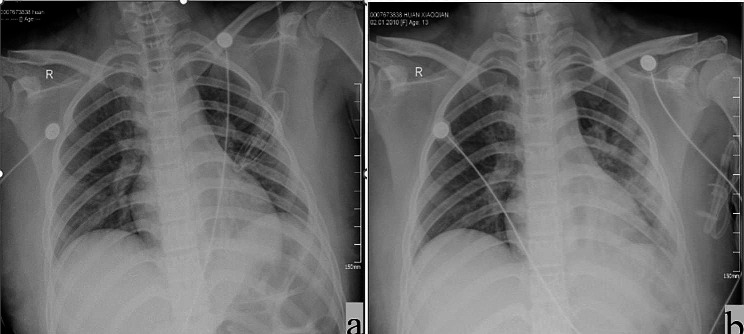
Chest radiograph results: a Case B on day 5 of admission, revealing no obvious abnormalities. b Case B on day 17 of admission, revealing obvious exudation from the left lung

**Fig. 3:**
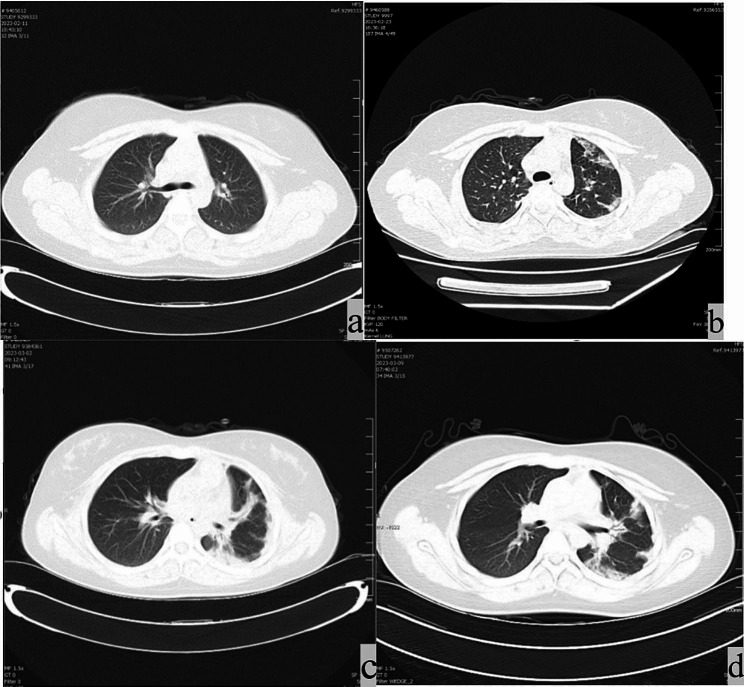
Chest CT results: **a** Case B on the second day of admission, revealing no obvious abnormalities. **b** Case B on day 13 of admission, revealing the interstitial exudation in both lungs, with the most pronounced effect for the left lung. **c** Case B on day 20 of admission, revealing the infection of both lungs with bronchial traction, dilation of the left lung, and bilateral pleural thickening. **d** Case B on day 27 of admission, revealing interstitial pneumonia in both lungs, slightly larger exudative lesions than in the previous film, with no apparent change in lesion range and a limited amount of pleural effusion on the left side; 2. Signs of anemia

Case B: A 13-year-old, previously healthy Chinese girl was sent to her local hospital 6 h after ingesting DQ. She had experienced nausea and vomiting, producing pale yellow vomitus with a pungent taste. She was flushed and experiencing chest tightness and an epigastric pain. She was taken to the local Emergency Department immediately after being found to have consumed the pesticide, and she exhibited normal vital signs when first evaluated. Her posterior pharynx was erythematous. After the administration of a gastric lavage, she was transferred to Lanzhou University Second Hospital PICU. Her vital signs on admission to our hospital were stable. Her initial laboratory findings, including complete blood count and biochemistry results, general status, clinical symptoms, DQ ingestion, and treatment are summarized in Table 1. The results of her laboratory studies on the first day of hospitalization are summarized in Table 2. Her imaging results can be seen in Figs. 1b and 2, and Fig. 3.

**Fig. 4:**
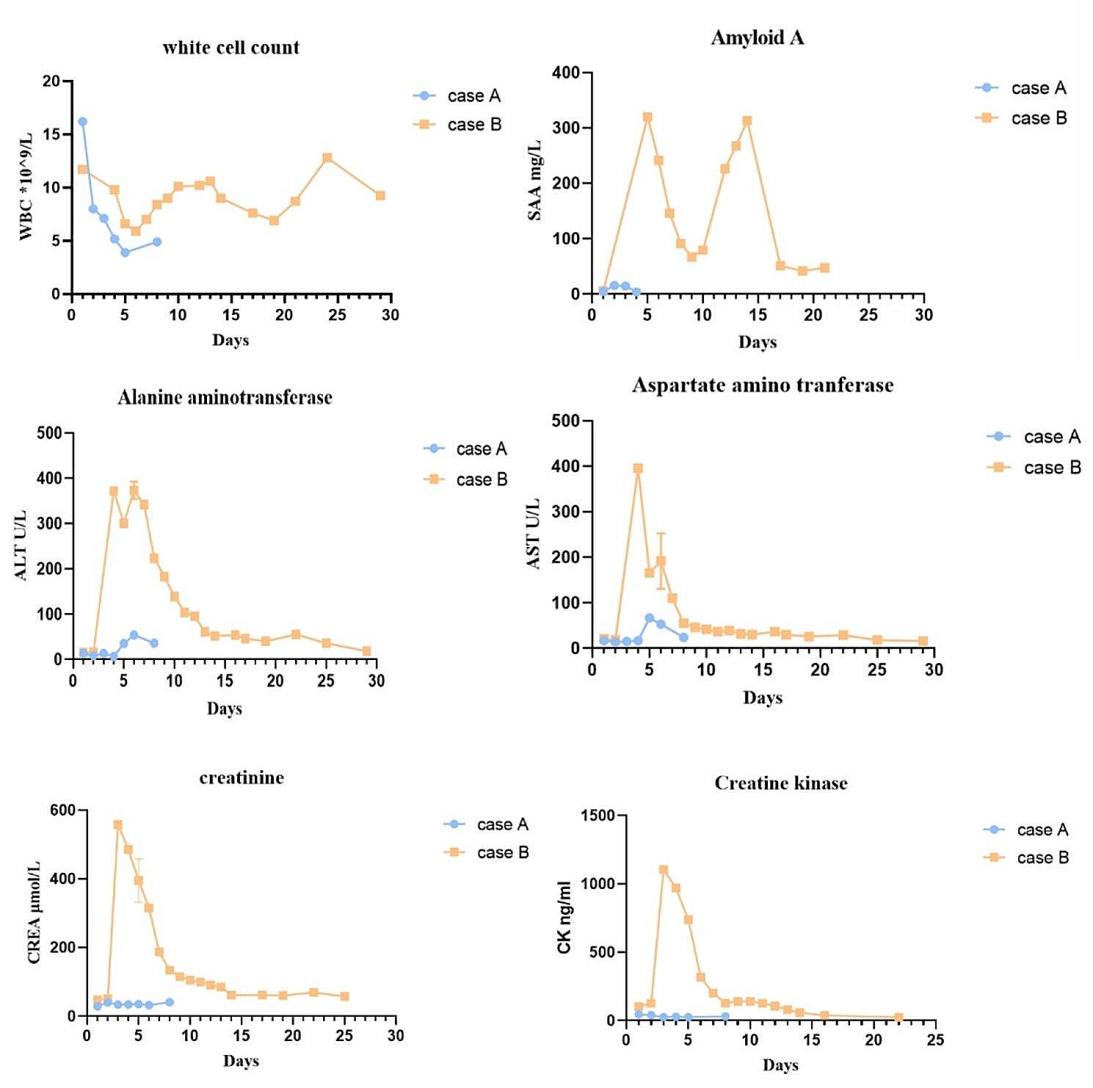
The above chart shows the dynamic changes in white blood cell counts, amyloid A, alanine aminotransferase, aspartate aminotransferase, creatinine, and creatine kinase levels after admission in these two children. The changes in laboratory indicators in child B were significantly changed compared with those in child A

In two cases, dynamic changes in laboratory results were seen in Fig. 4.

**Table 1:** The general situation, poisoning situation, clinical symptoms, and treatment scheme for each Case

Basic situation	Case A	Case B
General situation		
age	13 years	13 years
gender	girl	girl
weight	53 kg	65 kg
Basic diseases	none	none
DQ		
form(content, g/L)	liquid(200)	liquid(200)
way	Alimentary canal	Alimentary canal
category	oral、swallow	oral、swallow
dosage	About 15 ml	About 20 ml
clinical symptoms		
Burning sensation in oropharynx	√	√
nausea/vomit	√	√
abdominalgia/diarrhea	√	√
hematochezia	×	×
oropharyngeal/esophageal ulcer	√	√
Have a fever	×	×
cough	×	√
Short of breath	×	×
Chest tightness	×	√
jaundice	×	×
oliguria	×	×
anuria	×	×
dizziness	×	×
headache	×	×
spasm	×	×
coma	×	×
erythra	×	×
Pulmonary imaging changes	×	√
Treatment		
gastric lavage	√	√
adsorbent	×	√
drainage	×	√
diuretic	√	√
omeprazole	√	√
glucocorticoid	×	√
antibiotic	×	√
Vitamin C	√	√
glutathione	√	√
mecobalamin	×	√
CBP	√	√
CBP times	3	6
Interval time(h)		
Poisoning and first gastric lavage	1 h after	6 h after
Poisoning and first CBP	8 h after	90 h after
Hospitalized time	9d	29d
Discharge situation(outcome)	improve	improve

Note: DQ: diquat; CBP: continuous blood purification, using model HA320; √ stands for yes; × stands for non-existent/absent

**Table 2:** Initial Patient Laboratory Values

Case number	WBC (×10^9^ /L)	NEUT (×10^9^ /L)	HB (g/dl)	PLT (×10^9^ /L)	CRP (mg/L)	SAA (mg/L)	PCT (ng/ml)	NT-proBNP (pg/ml)
A	16.2	14.99	138	263	< 9.99	< 4.8	0.027	17
B	11.7	9.85	126	3.9	< 9.99	5.96	0.05	104
**Case** **number**	**ALT (U/L)**	**AST (U/L)**	**CREA(µmol/L)**	**Urea(mmol/L)**	**APTT (s)**	**CK(ng/ml)**	**CK-MB (ng/ml)**	**LDH** **(U/L)**
A	13	17	28.8	4.3	39.6	46	3	203
B	155	21	50.6	31.4	31.4	103	< 3	268.9

WBC: leukocyte; NEUT: neutrophil; HB: hemoglobin; PLT: platelet; CRP: C-reactive protein; SAA: serum amyloid A; PCT: procalcitonin; NT-proBNP: N-terminal pro-brain natriuretic peptide BUN: blood urea nitrogen; ALT: alanine aminotransferase; AST: aspartate aminotransferase; CREA: creatinine; Urea: blood urea nitrogen; APTT: activated partial thromboplastin time; CK: serum creatine kinase; CK-MB: creatine kinase isoenzyme. LDH: lactate dehydrogenase

## Discussion and conclusions

A review of the corresponding medical records revealed that these two children were similar in age, gender, and weight. Except for the timing of hemoperfusion, the treatment of both children was also similar. The blood picture, kidney and liver biochemical indexes of child B, all indicate that the index will be significantly abnormal after a period of DQ intake. Child B also exhibited evidence of lung injury detected via chest CT scans that were consistent with prior reports and in sharp contrast to the lungs of Child A, who received hemoperfusion at an earlier time point, with this likely accounting for this difference. The child had no obvious abnormalities in the B brain magnetic resonance imaging(MRI).

After timely hemoperfusion in child A, there was no obvious abnormality in laboratory indicators. When child B did not undergo hemoperfusion, laboratory indicators such as blood picture, liver function and kidney function indicators were significantly increased, and gradually recovered after treatment, and the chest effect also appeared delayed imaging abnormalities.The poison stays in the body for a long time and cause damage over time if not adequately removed. As shown in the Tables summarizing the clinical course of these patients, DQ can cause changes in blood parameters, liver damage, and kidney damage upon poisoning. The damage to the liver and kidneys in the child that underwent early continuous blood purification was significantly less severe than that for the other child, and this child also exhibited no apparent pulmonary symptoms.The child stays in the hospital for a shorter period of time and costs less. It has been mentioned in the literature, although hemoperfusion should be performed as soon as possible to prevent elevated levels of diquat toxicity in tissues [19].

Based on these results, we recommend that children suffering from DQ poisoning be given symptomatic supportive treatments such as gastric lavage, excretion, antioxidant administration, the removal of inflammatory mediators, and fluid replacement. In the case of rapid diquat poisoning, even in the absence of clear laboratory indicators, such as testing for poison dose and type, timely and early provision of renal supportive therapy (such as hemoperfusion) is also important to reduce multi-organ damage and shorten the length of hospital stay. The long-term prognosis of the child after treatment includes growth and intellectual development, and long-term follow-up is required.

**Declarations**.

## Data Availability

Data supporting our findings were taken from the patient’s folder.The datasets used and analysed during the current study are available from the corresponding author on reasonable request.

## Abbreviations

DQ: diquat
PICU: pediatric intensive care unit
CBP: continuous blood purification
WBC: leukocyte
NEUT: neutrophil
HB: hemoglobin
PLT: platelet
CRP: C-reactive protein
SAA: serum amyloid A
PCT: procalcitonin
NT-proBNP: N-terminal pro-brain natriuretic peptide
BUN: blood urea nitrogen
ALT: alanine aminotransferase
AST: aspartate aminotransferase
CREA: creatinine
Urea: blood urea nitrogen
APTT: activated partial thromboplastin time
CK: serum creatine kinase
CK-MB: creatine kinase isoenzyme
MRI: magnetic resonance imaging
LDH: lactate dehydrogenase
